# Hearing impairment in murine model of Down syndrome

**DOI:** 10.3389/fgene.2022.936128

**Published:** 2022-08-04

**Authors:** Guang-Di Chen, Li Li, Andrew McCall, Dalian Ding, Zhuo Xing, Y. Eugene Yu, Richard Salvi

**Affiliations:** ^1^ Center for Hearing and Deafness, University at Buffalo, Buffalo, NY, United States; ^2^ Optical Imaging and Analysis Facility, School of Dental Medicine, University at Buffalo, Buffalo, NY, United States; ^3^ The Children’s Guild Foundation Down Syndrome Research Program, Genetics and Genomics Program and Department of Cancer Genetics and Genomics, Roswell Park Comprehensive Cancer Center, Buffalo, NY, United States; ^4^ Genetics, Genomics and Bioinformatics Program, University of New York at Buffalo, Buffalo, NY, United States

**Keywords:** down syndrome, distortion product otoacoustic emissions, auditory brainstem response, conductive hearing loss, hair cells, cochlea, microCT

## Abstract

Hearing impairment is a cardinal feature of Down syndrome (DS), but its clinical manifestations have been attributed to multiple factors. Murine models could provide mechanistic insights on various causes of hearing loss in DS. To investigate mechanisms of hearing loss in DS in the absence of the cadherin 23 mutation, we backcrossed our DS mice, Dp(16)1Yey, onto normal-hearing CBA/J mice and evaluated their auditory function. Body weights of wild type (WT) and DS mice were similar at 3-months of age, but at 9-months, WT weighed 30% more than DS mice. Distortion product otoacoustic emissions (DPOAE), a test of sensory outer hair cell (OHC) function negatively impacted by conductive hearing loss, were reduced in amplitude and sensitivity across all frequencies in DS mice. The middle ear space in DS mice appeared normal with no evidence of infection. MicroCT structural imaging of DS temporal bones revealed a smaller tympanic membrane diameter, oval window, and middle ear space and localized thickening of the bony otic capsule, but no gross abnormalities of the middle ear ossicles. Histological analysis of the cochlear and vestibular sensory epithelium revealed a normal density of cochlear and vestibular hair cells; however, the cochlear basal membrane was approximately 0.6 mm shorter in DS than WT mice so that the total number of hair cells was greater in WT than DS mice. In DS mice, the early and late peaks in the auditory brainstem response (ABR), reflecting neural responses from the cochlear auditory nerve followed by subsequent neural centers in the brainstem, were reduced in amplitude and ABR thresholds were elevated to a similar degree across all frequencies, consistent with a conductive hearing impairment. The latency of the peaks in the ABR waveform were longer in DS than WT mice when compared at the same intensity; however, the latency delays disappeared when the data were compared at the same intensity above thresholds to compensate for the conductive hearing loss. Future studies using wideband tympanometry and absorbance together with detailed histological analysis of the middle ear could illuminate the nature of the conductive hearing impairment in DS mice.

## Introduction

The incidence of hearing impairment in patients who have Down syndrome (DS) is high (Keiser et al., 1981; Roizen et al., 1993; Laws and Hall, 2014) ranging from 34% to 78% in children (Shott et al., 2001; Yam et al., 2008; Raut et al., 2011). The nature of these hearing impairments is varied (Diefendorf et al., 1995). Many patients who have DS have conductive hearing losses in which sound transmission through the external ear and middle ear is reduced, resulting in a flat hearing loss. Conductive hearing losses can arise from craniofacial malformations of the external ear (Grundfast and Camilon, 1986; Diefendorf et al., 1995), while others are caused by fluid buildup in the middle ear (Schwartz and Schwartz, 1978; Austeng et al., 2013) or deformities that reduce the mobility of the middle ear ossicles (Balkany et al., 1979). Structural abnormalities have also been observed within the cochlea and sometimes the vestibular system (Krmpotic-Nemanic, 1970; Igarashi et al., 1977; Harada and Sando, 1981; Saliba et al., 2014). This may explain why up to 50% of adult patients with DS have been found to have sensorineural hearing loss (Glovsky, 1966; Brooks et al., 1972; De Schrijver et al., 2019). Balance problems are also associated with DS, but the extent to which these are associated with peripheral vestibular disorders versus central or motor disorders is unclear (Inagaki et al., 2011; Intrapiromkul et al., 2012; Villarroya et al., 2012; Clark et al., 2017; Capio et al., 2018). Abnormalities have also been reported in the central nervous system of patients with DS, such as reduced brain volume (Fujii et al., 2017; Rodrigues et al., 2019), stunted dendrite growth and atrophy (Becker et al., 1991) and abnormalities in the auditory brainstem response (ABR) (Squires et al., 1980; Folsom et al., 1983; Widen et al., 1987).

Auditory function has also been evaluated in murine models of DS in order to gain mechanistic insights into the disease. Ts65Dn and Dp1Tyb DS-mice were found to have mild to moderate flat hearing losses attributed to otitis media (Han et al., 2009; Bhutta et al., 2013; Lana-Elola et al., 2021). Many Ts65Dn mice presented with otitis media with effusion and inflammation of the middle ear, histological changes consistent with a conductive hearing loss (Han et al., 2009; Bhutta et al., 2013). In one study, chronic otitis media was also observed in Dp(16)1Yey mice, which have a duplication of the entire human chromosome 21 orthologous region on MMU16. However, otitis media was absent in other DS mouse models (Bhutta et al., 2013). Patients with DS show signs of premature aging (Horvath et al., 2015; Gensous et al., 2020), which may explain why some adults present with sensorineural hearing loss and early onset presbycusis by the second decade of life (Buchanan, 1990). However, we are unaware of any studies that have explicitly tested for early-onset sensorineural degeneration, cochlear malformation or central auditory dysfunction in adult DS mice. To address these issues, we first backcrossed our Dp(16)1Yey mice (Li et al., 2007) on to the CBA/J strain to eliminate the Cdh23 early-onset hearing loss mutant locus present in its strain background (Zheng et al., 1999; Johnson et al., 2017). Cochlear outer hair cell (OHC) sensory function was evaluated by recording distortion product otoacoustic emissions (DPOAEs); however, DPOAEs can also be depressed by conductive hearing loss (Hassmann et al., 1998; Gehr et al., 2004; Qin et al., 2010). The neural response from the cochlea and auditory brainstem were assessed by measuring the early and late components of the ABR. Afterwards, the auditory temporal bones and cochleae were harvested to test for hair cell loss in the cochlea and vestibular sensory epithelium. In some cases, we used microcomputed tomography (microCT) to test for gross structural abnormalities in the osseous structures of the temporal bone, middle ear capsule, ossicles, and cochlea.

## Materials and methods

### Subjects

The functional and anatomical studies were carried out on six male and nine female Dp(16)1Yey mice and nine male and seven female wild type (WT) mice (Li et al., 2007). Body weights of male and female mice were recorded prior to ABR testing. This mutant strain contains one duplication engineered between the Lipi gene and the Zbtb21 gene on mouse chromosome 16. The Dp(16)1Yey mice (Li et al., 2007) were backcrossed for at least two generations on CBA/J mice, a strain that shows little evidence of age-related hearing impairment until extremely late in life (Spongr et al., 1997; Zheng et al., 1999; Han et al., 2016). During backcrossing, the B6-specific age-related Cdh23c753A allele is converted to CBA/J specific Cdh23c753G, which was confirmed by sequencing (Kane et al., 2012) (Supplementary Figure S1).

### Distortion product otoacoustic emissions

DPOAEs were measured in order to assess the functional status of the outer hair cells in the cochlea; however, DPOAEs can also be negatively affected by conductive hearing loss. DPOAEs were measured in 6–8 month old mice as described previously (Liu et al., 2020; Ding et al., 2021). Mice were anesthetized with a mixture of ketamine (50 mg/kg, i.p.) and xylazine (6 mg/kg i.p.) and placed on a heating pad in a sound attenuating booth. An Extended-Bandwidth Acoustic Probe System (ER10X, Etymotic Research, Elk Grove Village, IL, United States), with two calibrated loudspeakers and a low noise microphone, was inserted into the right ear canal. Custom software (MatLab 6.1) was used to generate F1 and F2 (192 kHz sampling rate, 24-bit D/A converter); the F2/F1 ratio was set to 1.2, and the intensity of F2 (L2) was 10 dB lower than that of L1. Each stimulus was 90 ms in duration, presented at 5 Hz and repeated 32 times. The acoustic signal in the ear canal was measured with the low noise microphone. The microphone output was digitized with a sound card (RME Babyface Pro, 192 kHz sampling rate, 24-bit A/D converter), and the amplitudes of F1, F2, and 2F1–F2 were computed using a fast Fourier transform (FFT) within a frequency band of 12.5 Hz. The noise floor was measured in two 25 Hz bands surrounding 2F1–F2 (−25 to −50 and +25 to +50 Hz from 2F1–F2). DPOAE input/output (I/O) functions were constructed at F2 frequencies of 8, 16, 32, and 64 kHz by plotting the amplitude of 2F1–F2 as function of L2 intensity at each F2 frequency. L2 intensity was varied from 25 to 70 dB SPL in 5-dB SPL steps. DPOAE threshold was defined as the L2 level above which the DPOAE exceeded the mean noise floor by at least 5 dB.

### Auditory brainstem response

The ABR was recorded in 3 and 9-month-old mice as described previously (Chen et al., 2014; Ding et al., 2021). Mice were anesthetized with a mixture of ketamine (50 mg/kg, i.p.) and xylazine (6 mg/kg i.p.). Platinum subdermal needle electrodes (F-E2-24, Natus) were placed at the vertex (active), posterior bulla (reference), and behind the shoulder blade (ground) and the signals from the electrodes were amplified by a TDT Headstage-4 bio-amplifier. A TDT RX6 Multifunction Processor and a TDT RX5-2 Pentusa Base station running custom software (MatLab 6.1) were used to generate acoustic stimuli and record the ABR. Acoustic stimuli were calibrated with a ½″ microphone (model 2540, Larson Davis), a microphone preamplifier (model 2221, Larson Davis) and custom sound calibration software (MatLab 6.1).

Alternating phase tone bursts (5-ms duration, 1-ms rise/fall time, cosine^2^-gated), 4, 8, 16, and 32 kHz were generated at a sampling rate of 200 kHz. The tone bursts were presented through a MF1 loudspeaker (Tucker-Davis Technologies, Alachua, FL, United States) located 10 cm in front of the right ear. Stimuli were presented at a rate of 21.6/s and sound level was varied from 10 to 90 dB SPL in 10-dB SPL steps. Electrical responses were amplified 5020 times; bandpass filtered from 300 to 3,000 Hz, and averaged 800X from 90–70 dB SPL and 1,500 times from 60–10 dB SPL.

**FIGURE 1:**
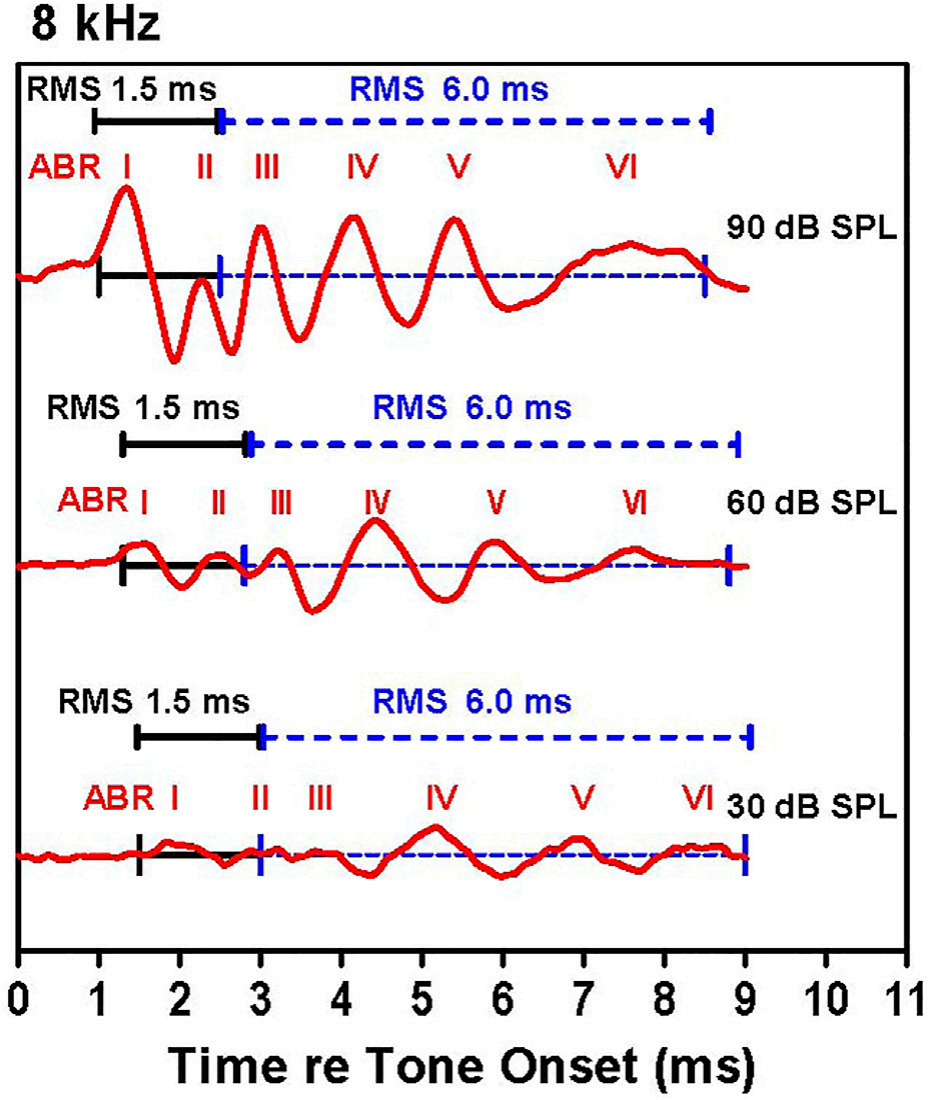
Examples of auditory brainstem response (ABR) waveforms elicited by 8 kHz tone bursts. The early (1.5 ms duration, black line) and late (6.0 ms duration, dashed blue line) RMS analyses windows are shown along with the major ABR peaks labeled I through VI. The early analysis window starts at the onset of wave I and continues for 1.5 ms; this is immediately followed by the 6.0 ms late analysis window. The latency of the ABR peaks increased as intensity decreased causing a slight rightward shift of the analysis windows as intensity decrease.

Our strategy for determining the amplitude and latency of the ABR is illustrated in Figure 1, which shows ABR waveforms at 30, 60, and 90 dB SPL with peaks labeled I, II, III, IV, V, and VI. The latencies of the various waves were measured from stimulus onset to the positive peak of each wave. The individual peaks became smaller as intensity decreased. Because the peaks also become wider as intensity decreases, we measured the root-mean-square (RMS) amplitude of the early and late portions of the response. The first RMS analysis window, computed over 1.5 ms beginning with onset of wave I, comprises the early response generated predominantly by the auditory nerve, hereafter referred to as wave I. Because the latency of wave I increased as intensity decreased, the analysis window shifted slightly rightward as intensity decreased. The second RMS analysis, computed over 6.0 ms starting at the end of the wave I analysis window, reflects neural activity largely generated proximal to the auditory nerve, hereafter referred to as the ABR response. The latencies, amplitudes and thresholds of the early wave I response and late ABR response were determined for each animal. ABR input/output functions were constructed at each frequency by plotting the RMS amplitudes of wave I and the ABR waveform as a function of intensity. Using individual input/output functions derived from the early wave I (1.5 ms) and late ABR (6 ms) analysis windows, wave I and ABR thresholds were defined as the intensity above which the RMS amplitude >0.1 µV.

### Cochlear histology and hair cells

Immediately after recording the final ABR, the anesthetized mouse was decapitated and the cochleae quickly removed and processed as previously described (Chen et al., 2000; Chen et al., 2009; Chen and Henderson, 2009). The round window and oval window were carefully opened and a small hole made in the apex of the cochlea to facilitate the initial perfusion. The cochleae were initially perfused with warm succinate dehydrogenase (SDH) staining solution containing 0.05% nitrotetrazolium blue chloride (^#^N6876, Sigma), 0.05 M sodium succinate, and 0.05 M phosphate buffer. Cochleae were then incubated in the solution for 1 h at 37°C. Afterwards, 10% buffered formalin was perfused through the cochleae. Afterwards, the cochlea were incubated in fixative for 2 days. The cochleae were subsequently decalcified in 7% EDTA (ethylenediaminetetraacetic acid) solution for 3–5 days. The entire length of the organ of Corti was then carefully dissected out as a flat surface preparation and examined over its entire length using a light microscope (DMBA300 Digital Microscope, Microscope World) to count the number of inner hair cells (IHCs) and outer hair cells (OHCs). The number of hair cells per 100 µm of cochlear length was plotted as a function of cochlear length in WT mice (*n* = 4) and DS mice (*n* = 6). Cochlear length was also measured in these WT and DS mice.

### Vestibular macular hair cells

To check for the possibility of histopathologies in the vestibular sensory epithelium, the temporal bones were harvested from WT (*n* = 3) normal and DS (*n* = 3) mice and evaluated using procedures described previously (Johnson et al., 2010; Ding et al., 2016). The anesthetized mice were decapitated, the inner ear carefully removed, the round and oval windows opened and 10% buffered formalin perfused though the openings. The temporal bones were then immersed in 10% buffered formalin for 24 h. After rinsing three times with 0.1 M PBS, the maculae of the utricle and saccule were carefully dissected out as a surface preparation, stained with Harris hematoxylin solution, mounted in glycerin on glass slides and cover slipped. The vestibular surface preparation of the maculae of the utricle and saccule were photographed with a digital camera (SPOT Insight, Diagnostic Instruments Inc.) attached to a Zeiss Axioskop microscope, processed with imaging software (SPOT Software, version 4.6) and Adobe Photoshop 5.5.

### MicroCT analysis of temporal bones

After completing the physiological recording, the anesthetized mice were decapitated and the temporal bones of WT (*n* = 2) and DS (*n* = 4) mice were removed from the skull. The middle ear space was gently opened by removing the tympanic membrane and then 10% buffered formalin was perfused into the middle ear space. Afterwards, the temporal bone containing the cochlea was placed in a small bottle containing 10% buffered formalin and the sample stored at 4°C. Several weeks later, the temporal bones were washed with PBS and placed in a small glass container filled with PBS. The samples were placed in a ScanCo µCT 100 scanner (SCANCO Medical, Brüttisellen, Switzerland) and scanned with the following parameters: 70 kVp, 114 μA, 375 ms exposure, 5-µm nominal isotropic resolution.

CT images were analyzed using FIJI image analysis software (Schindelin et al., 2012). Image masks were created using 3D ImageJ Suite simple segmentation with a calibrated bone density cutoff of 350 mg hydroxyapatite/cm^3^ and a 625,000 µm^3^ (5,000 voxels) minimum size (Ollion et al., 2013). Masks were then applied to the original data to isolate the bones from background signal. To facilitate side-by-side comparisons, all images were then transformed *via* image registration using the Fijiyama plugin to the same reference bone (Fernandez and Moisy, 2021). Prior to registration, all left-sided temporal bones were mirrored so they could align with their right-sided counterparts. Then, a rough manual registration was performed, followed by automatic rigid registration *via* block matching with default parameters. 3D rendering and animation was conducted using the 3Dscript plugin (Schmid et al., 2019).

The overall length of the temporal bone was determined by taking the Feret’s diameter from 3D ImageJ Suite. Measurements of the tympanic cavity were taken from the same z-reference cross-section following image registration. The three measurements of the tympanic cavity taken were the shortest distance across the opening, the shortest distance across the approximate middle of the cavity, where a bony ridge is located; and the depth of the cavity, starting at the midpoint of the measurement line across the opening, and passing through the midpoint of the middle line. Lastly, the shortest distance across the oval window was measured. Prior to this measurement, a small volume around the oval window was isolated (∼0.5 mm^3^) and an additional automatic rigid registration was performed on this smaller region.

### Analyses

Graphs were prepared with GraphPad Prism (version 5) and statistical analyses were performed with GraphPad Prism (version 5) or SigmaStat software (version 3.5) as described below.

## Results

### Body weight

**FIGURE 2:**
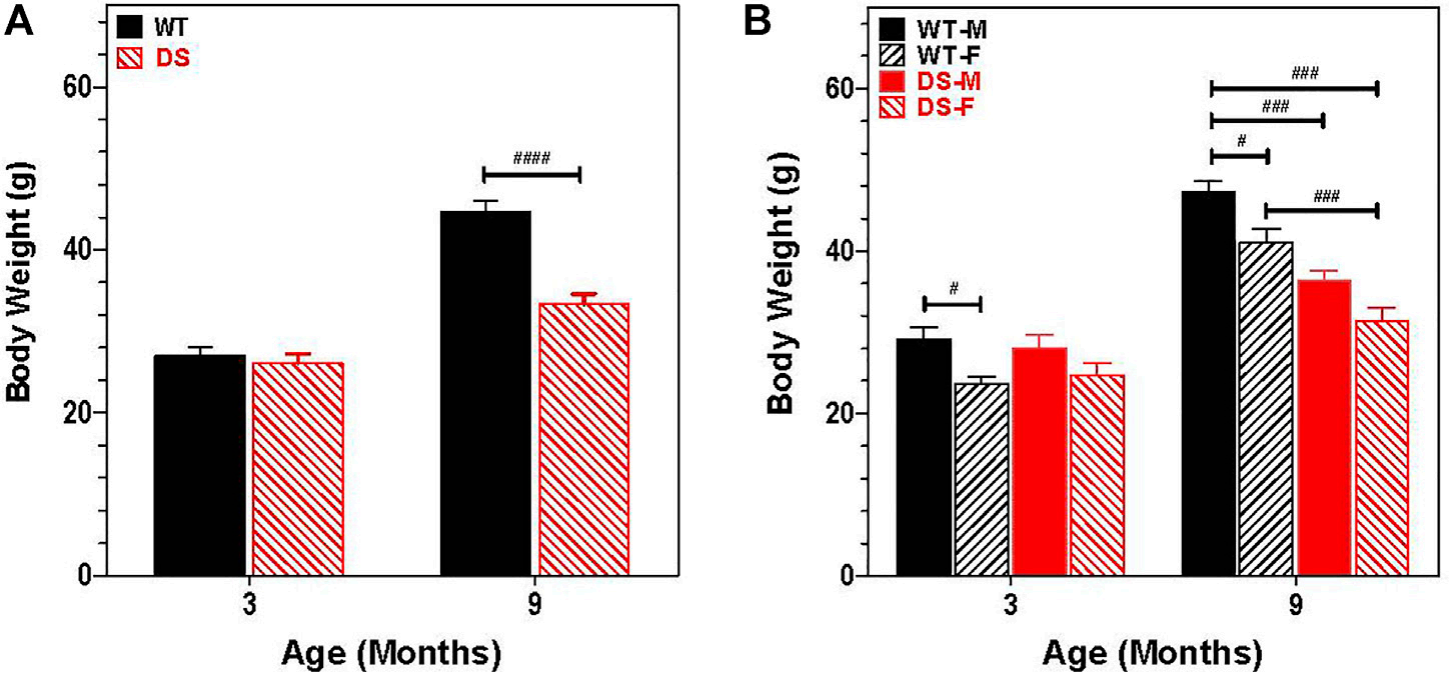
Increase in body weight is age and gender dependent. **(A)** Increases in body weight between 3 and 9 months of age is significantly less in DS mice than WT. Mean body weight (g) (+SEM) of WT and DS mice similar at 3 months of age, but at 9 months of age, DS mice weigh significantly less than WT mice. ^####^ *p* < 0.0001). **(B)** Increase in body weight between 3 and 9 months of age is gender dependent. Two way repeated measure analysis of variance revealed a significant effect of genotype (F 3, 26 = 10.35, *p* < 0.0001; age (F 1, 26 = 599.49, *p* < 0.0001) and interaction of age x genotype, F 3, 26 = 37.06, *p* < 0.0001, other significant group differences identified by Bonferroni post-hoc analysis indicated in figure, ^#^ *p* < 0.05, ^##^ *p* < 0.01, ^###^ *p* < 0.001.

Growth retardation and short stature are cardinal features of humans with DS (Cronk et al., 1988; Myrelid et al., 2002; Styles et al., 2002). Therefore, we recorded the body weights of 30 mice at 3 and 9 months of age (six male DS, nine female DS, nine male WT and six female WT). Figure 2A shows the change in weight with age for WT and DS mice of both gender. Body weight increased with age, but the increase was greater in WT than DS mice. At 3 months of age, DS and WT mice weighed approximately 26.5 g, but at 9 months of age, the mean weight of WT mice was approximately 45 g versus 33 g in DS mice. To identify potential gender differences, body weights of males and females were compared at 3- and 9-months of age (Figure 2B). Body weights increased with age, but the increase was greater for males than females. A two way repeated measure analysis of variance (ANOVA) revealed a significant effect of age (F 1, 26 = 599.49, *p* < 0.0001), a significant effect of genotype (F 3, 26 = 10.35, *p* < 0.0001) and a significant age x gender-genotype interaction (F 3, 26 = 37.06, *p* < 0.0001). Bonferroni post-hoc tests revealed significant differences between WT male and WT female mice (*p* < 0.05) at 3-months and 9-months of age, between WT males and DS males at 9-months of age (*p* < 0.001), between WT males and DS females at 9-months of age (*p* < 0.001) and between WT females and DS females at 9-months of age (*p* < 0.001).

### Distortion product otoacoustic emissions

**FIGURE 3:**
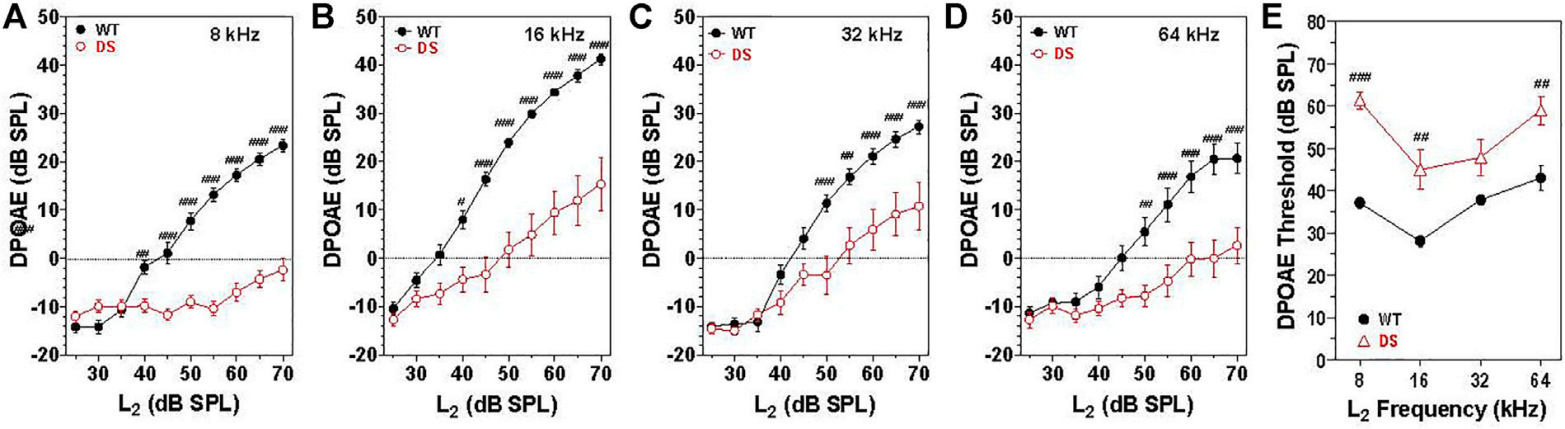
DPOAE amplitudes significantly less in DS mice (*n* = 15) compared to WT mice (*n* = 15). Mean (+/-SEM) DPOAE input/output functions. Amplitude plotted as a function of L2 intensity at F2 frequencies of **(A)** 8, **(B)** 16, **(C)** 32, and **(D)** 64 kHz. **(E)** DPOAE thresholds significantly greater in DS mice versus WT mice. DPOAE threshold versus frequency (*n* = 15, +/− SEM). ^#^ *p* < 0.05; ^##^ *p* < 0.01; ^###^ *p* < 0.001.

Sound transmitted through the middle ear enters the cochlea where it generates a traveling wave that propagates in a tonotopic manner along the basilar membrane stimulating the OHCs and IHCs. The OHC electromotile response (Brownell, 1990) enhances the motion of the basilar membrane. However, in doing so, it generates distortion products, which are transmitted in the reverse direction back through the cochlea, middle ear and into the external ear where the distortion sound can be detected with a microphone (Brown and Kemp, 1984; Trautwein et al., 1996; Liberman et al., 2002). To assess the integrity of the OHCs as well as forward and reverse sound transmission through the middle ear, mean DPOAE input/output functions were constructed from WT (*n* = 15) and DS (*n* = 15) mice at F2 frequencies of 8, 16, 32, and 64 kHz (Figures 3A–D). These frequencies were selected to cover the range of hearing in mice (Muller et al., 2005). Mean DPOAE amplitudes in WT mice started to increase above the noise floor at L2 intensities between 30 and 40 dB SPL. Mean DPOAE amplitudes increased with L2 stimulus intensity reaching maximum levels around 24, 42, 27, and 28 dB SPL at 8, 16, 32, and 64 kHz respectively. Mean DPOAE input/output functions from DS mice were shifted to the right of the WT mice by approximately 25, 15, 15, and 15 dB at 8, 16, 32, and 62 kHz respectively. Mean DPOAE amplitudes in DS mice increased with intensity reaching maximum levels of −2, 15, 10, and 4 dB SPL at 8, 16, 32, and 64 kHz respectively.

At 8 kHz, DPOAE amplitudes were significantly less in DS mice than WT mice at L2 intensities from 40 to 70 dB SPL [two-way repeated measure ANOVA, significant effect of intensity (F 9, 252 = 87.3, *p* < 0.0001), genotype (F 1, 252 = 96.8, *p* = 0.0037) and intensity x genotype (F 9, 252 = 44.1, *p* < 0.001), Bonferroni post-hoc *p* < 0.001 from 45 to 70 dB SPL, *p* < 0.001 at 40 dB SPL]. Mean DPOAE amplitudes at 16 kHz were significantly less in DS mice than WT mice from 40 to 70 dB SPL [two-way repeated measure ANOVA, significant effect of intensity (F 9, 252 = 137.9, *p* < 0.0001), genotype (F 1, 252 = 27.0, *p* < 0.0001), and intensity x genotype (F 9, 252 = 16.0, *p* < 0.001), Bonferroni post-hoc *p* < 0.001 from 45 to 70 dB SPL, *p* < 0.05 at 40 dB SPL]. At 32 kHz, DPOAE amplitudes were significantly less in DS than WT mice at L2 intensities from 50 to 70 dB SPL [two-way repeated measure ANOVA, significant effect of intensity (F 9, 252 = 112.2, *p* < 0.0001), genotype (F 1, 252 = 10.0, *p* = 0.004) and intensity x genotype (F 9, 252 = 8.34, *p* < 0.0001), Bonferroni post-hoc *p* < 0.001 from 50 to 70 dB SPL, *p* < 0.001 at 55 dB SPL]. At 64 kHz, DPOAE amplitudes were significantly less in DS than WT mice at L2 intensities from 50 to 70 dB SPL [two-way repeated measure ANOVA, significant effect of intensity (F 9, 252 = 40.1, *p* < 0.0001), genotype (F 1, 252 = 15.6, *p* = 0.0005) and intensity x genotype (F 9, 252 = 7.0, *p* < 0.0001), Bonferroni post-hoc *p* < 0.001 from 55–70 dB SPL and *p* < 0.05 at 50 dB SPL].

Mean (+/− SEM) DPOAE thresholds in WT mice (*n* = 15) ranged from 28 dB at 16 kHz to 43 dB at 64 kHz (Figure 3E) whereas the mean (+/− SEM) DPOAE thresholds in DS mice (*n* = 15) varied from 45 dB at 16 kHz to 61 dB at 8 kHz. DPOAE thresholds were 11–24 dB higher in DS than WT mice. Thresholds in DS mice were significantly higher than those in WT mice at 8, 16, and 64 kHz [two-way repeated measure ANOVA, significant effect of frequency (F 3, 84 = 21.05, *p* = 0.0001), genotype (F 1, 84 = 25.03, *p* < 0.0001) and frequency x genotype (F 3, 84 = 4.11, *p* < 0.009), Bonferroni post-hoc (*p* < 0.001 at 8 and 16 kHz; *p* < 0.01 at 64 kHz].

### Wave I and auditory brainstem response thresholds

**FIGURE 4:**
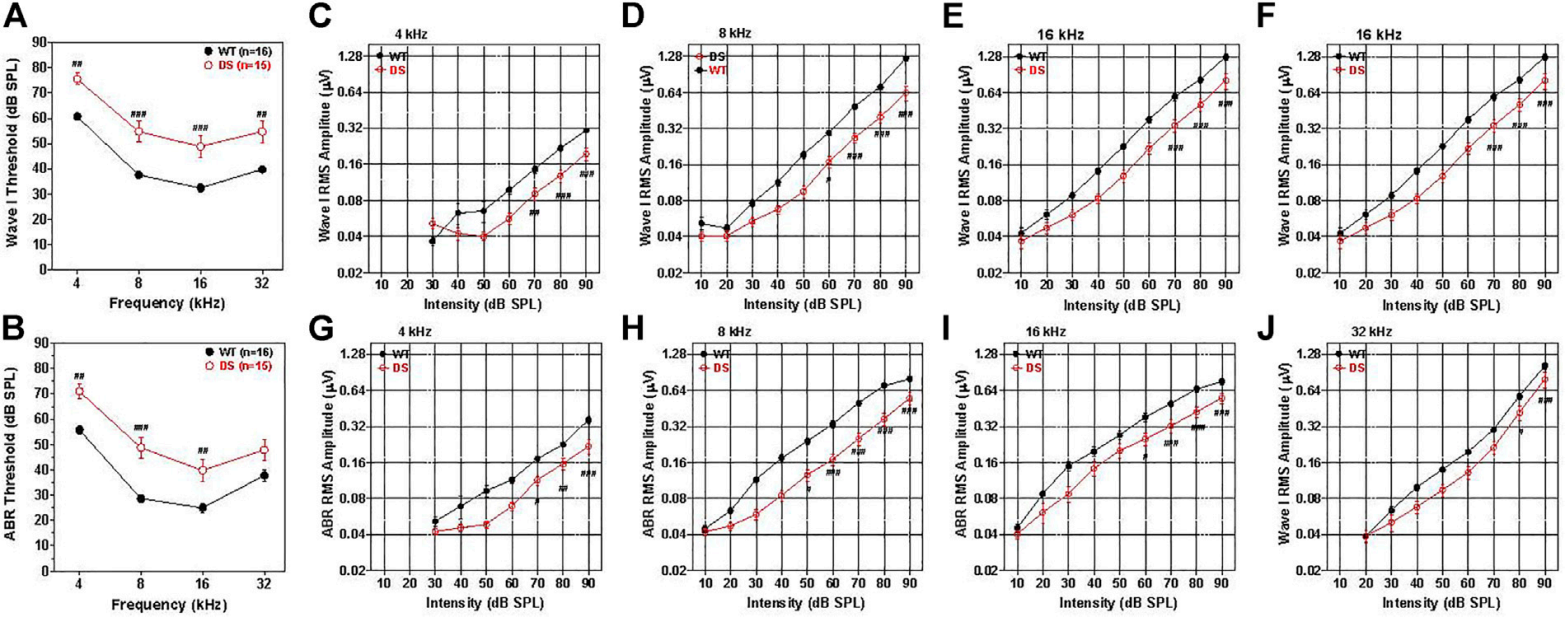
Wave I and ABR thresholds are 15–20 dB higher in DS mice than WT mice. **(A)** Mean (+/− SEM) wave I thresholds versus frequency in DS (*n* = 15) and WT (*n* = 16) mice. Wave I thresholds significantly higher in DS than WT mice at 4, 8, 16, and 32 kHz **(B)** Mean (+/− SEM) ABR thresholds in DS mice (*n* = 15) significantly higher than WT mice at 4, 8, and 16 kHz (^###^ *p* < 0.001, ^##^ *p* < 0.01). Wave I and ABR amplitudes are smaller in DS than WT mice. **(C–F)** Mean WT (*n* = 16, +/− SEM) and DS (+/− SEM, *n* = 15) wave I RMS amplitude (log base 2 scale) versus intensity functions at 4, 8, 16, and 32 kHz. **(G–J)** Mean WT (*n* = 16, +/− SEM) and DS (+/− SEM, *n* = 15) ABR RMS amplitude (log base 2 scale) versus intensity at 4, 8, 16, and 32 kHz. Two way repeated measure analysis of variance. Significant differences between DS and WT mice indicated in each panel: ^#^ *p* < 0.05, ^##^ *p* < 0.01, ^###^ *p* < 0.001.

ABR thresholds have previously been reported in DS mice, but these were measured in mice developed on a background strain with early age-related hearing loss (Lana-Elola et al., 2021). To avoid this problem, we backcrossed our DS mice onto the CBA/J strain thus eliminating this potential confounder. In WT mice, the mean (+/− SEM, *n* = 16) wave I thresholds, reflecting the neural output of the cochlea, were approximately 35 dB SPL at 16 kHz, 40 dB SPL at 8 and 32 kHz and 60 dB SPL at 4 kHz (Figure 4A). The mean wave I ABR thresholds in the DS mice (+/− SEM, *n* = 15) paralleled those in the WT mice, but were 15–17 dB higher at 4, 8, 16, and 32 kHz. A two-way ANOVA revealed a significant main effect of genotype [F (1, 87) = 19.98, *p* < 0.0001] and a significant main effect of frequency [F (3, 87) = 129.98, *p* < 0.0001]. A Bonferroni post-hoc analysis indicated that wave one thresholds were significantly higher in DS than WT mice at 4 kHz (*p* < 0.01), 8 kHz (*p* < 0.001), 16 kHz (*p* < 0.001) and 32 kHz (*p* < 0.01). Thus, wave I measures revealed a nearly parallel threshold upshift of 15–17 dB across frequency in DS mice suggestive of a conductive hearing loss.

The mean ABR thresholds in WT mice, reflecting brainstem neural activity, were approximately 25 dB SPL at 16 kHz, 30 dB SPL at 8 kHz, 40 dB SPL at 32 kHz and 57 dB SPL at 4 kHz (Figure 4B). The ABR thresholds were generally 5–7 dB lower than wave I thresholds, likely due to greater neural synchrony needed to elicit wave I responses (Petoe et al., 2010). Mean (+/− SEM, *n* = 15) ABR thresholds in DS and WT mice were largely parallele to one another, but the thresholds were roughly 15, 20, 14, and 10 dB higher in DS than WT mice at 4, 8, 16, and 32 kHz respectively. A two-way repeated measures ANOVA revealed a significant main effect of genotype [F (1, 87) = 16.33, *p* = 0,004], a significant main effect of frequency [F (3, 87) = 135.51, *p* < 0.0001] and a significant interaction of frequency x genotype [F (3, 87) = 3.17, *p* = 0.284].

### Wave I and auditory brainstem response amplitudes

Wave I amplitudes were measured over a range of intensities up to 90 dB SPL and the data used to construct RMS amplitude-intensity functions at 4, 8, 16, and 32 kHz. The mean (+/− SEM) wave I amplitudes represented on a log base two scale are plotted as a function of intensity at 4, 8, 16, and 32 kHz in Figures 4C–F, respectively. The mean amplitudes increased monotonically as intensity increased except at low intensities at 4 and 8 kHz where responses were near threshold. Wave I amplitude-intensity functions in WT mice were similar at 8, 16, and 32 kHz, but amplitudes were substantially smaller at 4 kHz than at higher frequencies. Wave I amplitude-intensity functions in DS mice largely paralleled those in WT mice, but the input/output functions were shifted to the right of the WT functions by approximately 11–14 dB SPL. Wave I amplitudes at 4 kHz were significantly smaller in DS than WT mice at 70, 80, and 90 dB SPL (two-way repeated measured ANOVA, significant effect of intensity, [F (6, 174) = 114.41, *p* < 0.0001], genotype, [F (1, 174) = 27.43, *p* < 0.0001], genotype x intensity,[F (6, 174) = 8.62, *p* < 0.001], Bonferroni post hoc, (*p* < 0.01 at 70 dB, *p* < 0.001 at 80 and 90 dB SPL). At 8 kHz, wave I amplitude were significantly smaller in DS mice than WT mice from 60 to 90 dB SPL (two-way repeated measure ANOVA, significant effect of intensity (F (8, 232) = 230.58, *p* < 0.0001), genotype [F (1, 233) = 40.86, *p* < 0.0001], intensity x genotype [F = (8, 232) = 24.38, *p* < 0.001], Bonferroni post-hoc, (*p* < 0.05 at 60 dB SPL, *p* < 0.0001 at 70, 80 and 90 dB SPL). At 16 kHz, wave I amplitudes were significantly smaller in DS than WT mice at 70, 80, and 90 dB SPL (two-way repeated measure ANOVA, significant effect of intensity [F (8, 32) = 193.83, *p* < 0.0001], genotype [F (1, 232) = 18.73, *p* = 0.0002], intensity x genotype [F (8, 232) = 10.35, *p* < 0.0001], Bonferroni post-hoc, (*p* < 0.001 from 70–90 dB SPL). Wave I amplitudes at 32 kHz were significantly smaller in DS than WT mice at 80 and 90 dB SPL (two-way repeated measure ANOVA, significant effect of intensity [F (7, 203) = 154.68, *p* < 0.0001], genotype [F (1, 203) = 7.44, *p* < 0.0107], intensity x genotype [F (7, 203) = 2.79, *p* < 0.0087], Bonferroni post-hoc, (*p* < 0.05 at 80 dB SPL, *p* < 0.0001 at 90 dB SPL). The rightward shifts of the wave I input/output functions at 4, 8, 16, and 32 kHz are approximately the same magnitude as the wave I threshold shifts at these frequencies (Figure 4A) suggestive of a conductive hearing loss. Because the DS input/output functions are shifted to the right of the WT functions X-dB, increasing the stimulus intensity by X-dB would make the amplitude in DS mice approximately equal to those in WT mice.

To test for functional changes in the auditory brainstem, we compared the mean ABR amplitude-intensity functions of DS and WT mice at 4, 8, 16, and 32 kHz. Figures 4G–J shows the mean (+/− SEM) ABR amplitudes on a log scale versus intensity at 4, 8, 16, and 32 kHz. The mean amplitudes of WT and DS mice increased with intensity, but ABR amplitudes were smaller in DS than WT mice. The ABR amplitude-intensity functions of DS mice were shifted to the right of the WT mice approximately 10, 20, 15, and 10 dB at 4, 8, 16, and 32 kHz respectively, similar to the DPOAE thresholds shifts (Figure 3E). ABR amplitudes at 4 kHz were significantly less in DS mice than WT mice at 70, 80, and 90 dB SPL (two-way repeated measure ANOVA, significant effect of intensity [F 6, 174 = 109.77, *p* < 0.0001], genotype [F 1, 174 = 32.33, *p* < 0.001], intensity x genotype [F 6, 174 = 6.68, *p* < 0.0001], Bonferroni post-hoc, (*p* < 0.05 at 70 dB SPL, *p* < 0.01 at 80 dB SPL, *p* < 0.0001 at 90 dB SPL). ABR amplitudes at 8 kHz were significantly smaller in DS than WT mice from 50 to 90 dB SPL (two-way repeated-measure ANOVA, significant effect of intensity (F 8, 232 = 261.94, *p* < 0.0001), genotype (F 1, 232 = 31.67, *p* < 0.0001), intensity x genotype (F 8, 232 = 17.21, *p* < 0.0001), Bonferroni post-hoc, (*p* < 0.05 at 50 dB SPL, *p* < 0.001 at 60–90 dB SPL). ABR amplitudes at 16 kHz were significantly smaller in DS than WT mice from 60–90 dB SPL (two-way ANOVA, significant effect of intensity (F 8, 232 = 240.98, *p* < 0.0001), genotype (F 1, 232 = 11.51, *p* = 0.002), intensity x genotype (F 8, 232 = 8.84, *p* < 0.0001), Bonferroni post-hoc, (*p* < 0.05 at 60 dB SPL, *p* < 0.0001 from 70–90 dB SPL). At 32 kHz, ABR amplitudes were significantly less in DS than WT mice at 90 dB SPL (two-way repeated measure ANOVA, significant effect of intensity (F 7, 203 = 153.94, *p* < 0.0001), genotype (F 1, 203 = 5.85, *p* < 0.022), intensity x genotype (F 7, 203 = 3.19, *p* < 0.003), Bonferroni post-hoc, *p* < 0.0001 at 90 dB SPL). Because DS input/output functions are shifted to the right of the WT functions by approximately 10–20 dB, increasing the stimulus intensity by 10–20 dB would increase the amplitudes in DS-mice to approximately the same amplitudes as those of WT-mice.

### Wave I–V latencies

**FIGURE 5:**
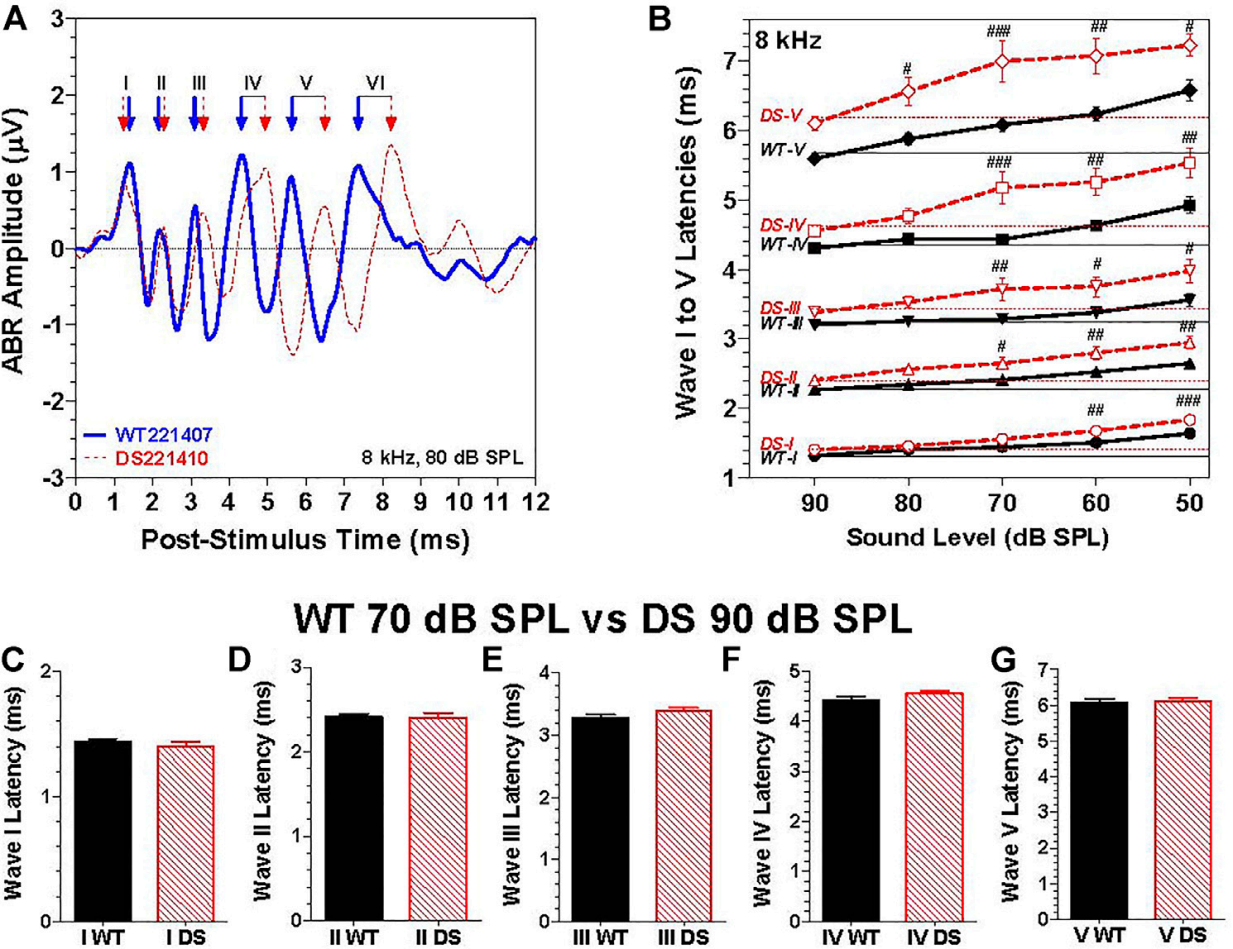
The latencies of waves I through V were longer in DS mice than WT mice at the same intensity. **(A)** Representative ABR waveforms from a WT (blue solid line) and a DS (dashed red line) mouse evoked by 8 kHz tone bursts presented at 80 dB SPL. Positive peaks of ABR wave I, II, III, IV, V, and VI indicated for WT (blue arrows) and DS (red arrows) mice. **(B)** Mean (+/- SEM) latencies of ABR waves I, II, III, IV and V of DS mice (red, dashed line) and WT mice (black, solid line) decreased as intensity increased from 50 to 90 dB SPL (note: intensity axis reversed). Mean latencies were shorter for DS than WT mice; the latency differences between DS and WT mice increased as intensity decreased. At each intensity, the latency increased from wave I to wave V. **(C–G)**. Mean (+SEM) wave I–V latencies of WT and DS mice are compared at 70 dB SPL versus 90 dB SPL respectively to compensate for ∼20 dB higher 8 kHz-thresholds in DS mice relative to WT mice. Latencies of wave I through V measured at the same dB level above threshold in DS and WT mice were nearly identical.

To evaluate neural transmission time in the auditory brainstem, we compared the post-stimulus time latencies of waves I through V elicited by 8 kHz tone bursts. The 8 kHz tone burst at 80 dB SPL evoked six well-defined peak in the ABR as illustrated by the waveforms from typical WT (Figure 5A, blue solid line) and DS mice (Figure 5A, dashed red line). The positive peaks of waves I through VI in the WT and DS mouse are indicated by blue and red arrows. Except for wave I, the latencies become progressively longer for waves II through VI. Results similar to this were obtained at other intensities and frequencies. To quantify the results, we measured the latencies of the positive peaks of wave I through V in each WT and DS mice for 8 kHz tone bursts presented at intensities from 50 to 90 dB SPL (Figure 5B). The mean (+/− SEM) latencies of waves I though V decreased as intensity increased from 50 to 90 dB SPL (Figure 5B, intensity axis reversed). The mean latencies of waves I through V were longer in DS than WT mice; these genotype latency differences were more pronounced at lower intensities and for the later peaks. The intensities at which the latencies of wave I, II, III, IV, and V were significantly longer in DS mice than WT mice are indicated in Figure 5B [Two-way repeated measure ANOVA; wave-I (F 1, 134 = 31.9, *p* < 0.0001), wave-II (F 1, 134 = 37.5, *p* < 0.0001), wave-III (F 1, 134 = 32.1, *p* < 0.0001), wave-IV (F 1, 134 = 42.2, *p* < 0.0001) and wave-V (F 1, 134 = 51.1, *p* < 0.0001). Bonferroni post-hoc tests, significant differences indicated in Figure 5B, ^#^ *p* < 0.05, ^##^: *p* < 0.01, ^###^: *p* < 0.001].

Because wave I through V latencies decrease with intensity, the latency differences between DS and WT mice, as exemplified by the data in Figure 5B, could simply be due to the fact that the 8-kHz ABR threshold is approximately 20 dB higher in DS than WT mice. To compensate for this difference in threshold, we compared the ABR latencies evoked by 8 kHz tone bursts presented at 90 dB SPL in DS mice versus 70 dB SPL in WT mice, i.e., at approximately the same intensity above threshold in each genotypes. The mean (+SEM) latencies of wave I, II, III, IV, and V in DS and WT mice were nearly identical (Figures 5C–G). Thus, the longer latencies in DS versus WT mice in Figures 5A,B are likely due to the 20 dB difference in thresholds due to a conductive hearing loss. To rule out the possibility of a sensory pathology in the cochlea, we compared the auditory hair cell densities in the cochlea and vestibular sensory epithelia.

### Cochlear and vestibular hair cells

**FIGURE 6:**
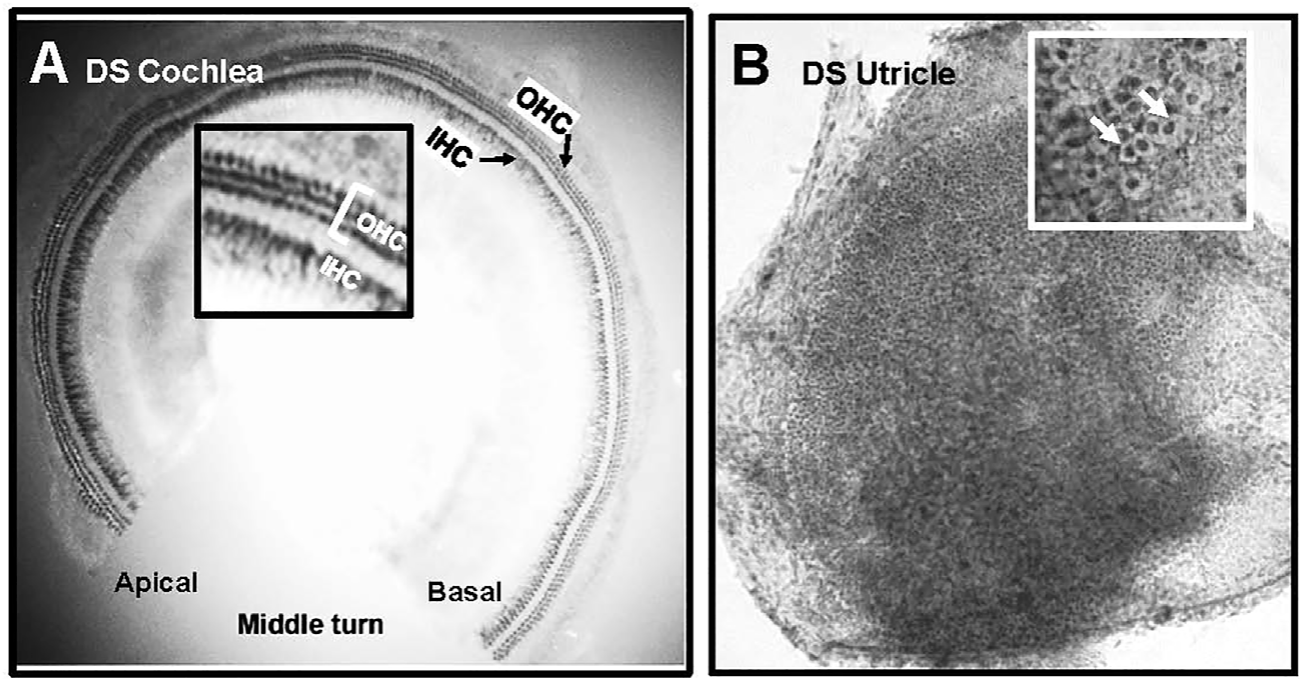
Normal population of hair cells in sensory epithelium of the cochlea and vestibular system of Down syndrome (DS) mice. Representative photomicrographs of **(A)** middle turn of DS mouse cochlea stained with succinate dehydrogenase. Three rows of outer hair cells (OHCs) and single row of inner hair cells (IHCs) spiral from the basal toward the apical region of the cochlea. Inset shows higher magnification view of three rows of OHCs and row of IHCs. **(B)** Representative photomicrograph of macula of utricle stained with Harris hematoxylin. Macula densely packed with vestibular hair cells. Inset shows higher magnification view of darkly stained hair cells surrounded by translucent chalice surrounding afferent nerve fibers (arrows).

The cochleae of 4 WT and 6 DS mice were stained with succinate dehydrogenase which strongly labels the numerous mitochondria present OHCs and IHCs. Figure 6A shows a representative photomicrograph of a surface preparation from the middle turn of a DS mouse with morphological features similar to those of normal WT cochleae (data not shown). Three parallel rows of OHCs and a single row of IHCs spiral from the basal toward the apical edge of the middle turn of the cochlea. The inset in the figure shows a higher magnification view of the three rows of OHCs and single row of IHCs. The OHCs and IHCs formed continuous rows indicating that the cochlear sensory hair cells were intact in DS mice.

Figure 6B shows a representative photomicrograph of a surface preparation of the macula of the utricle of a DS mouse stained with Harris hematoxylin. The sensory epithelium appeared normal and homogenously packed with vestibular hair cells. The inset in the figure shows a higher magnification view of the sensory epithelium, which is characterized by round, darkly stained vestibular hair cells surrounded by a large, translucent region representing the afferent terminal that envelops the base and lateral wall of type I vestibular hair cells. The sensory hair cells in the maculae of the utricle and saccule of DS mice appeared normal and similar to those of WT mice. No gross differences in vestibular hair cell density were observed between DS and WT mice.

**FIGURE 7:**
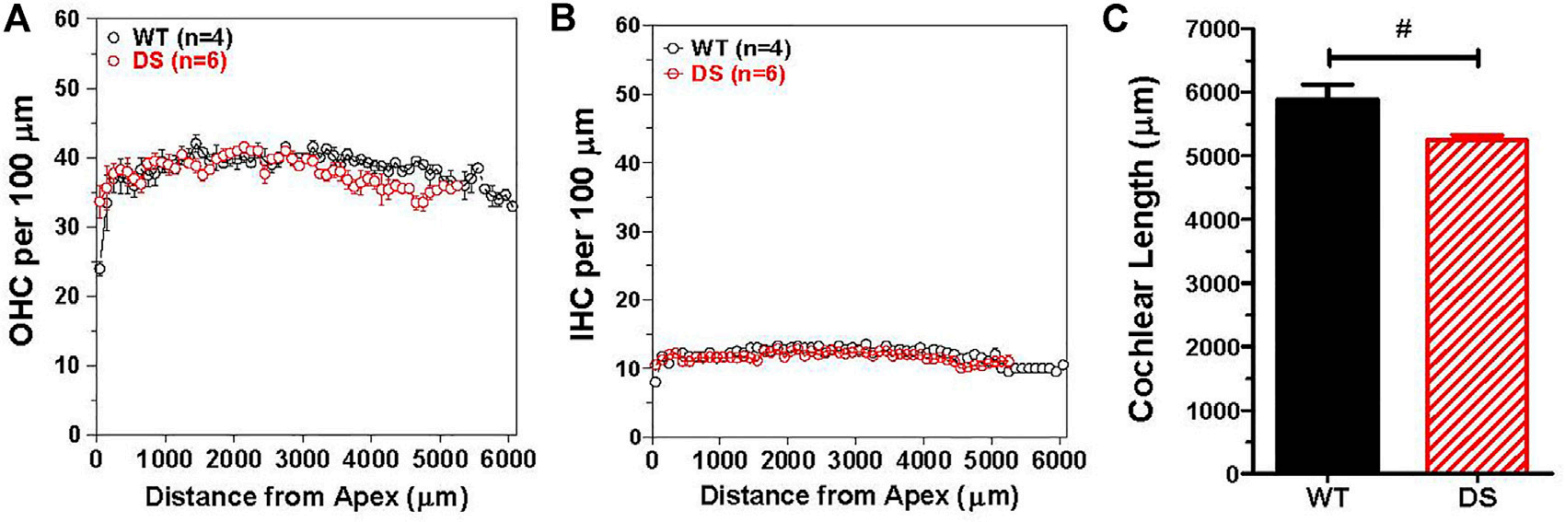
OHC and IHC densities in DS mice are similar to WT mice, but length of cochlea is significantly longer in WT mice than DS mice. Mean (+/− SEM) numbers of **(A)** OHCs and **(B)** IHCs in 100 μm intervals as a function of distance from the apex of the cochlea in DS (*n* = 6) and WT (*n* = 4) mice. Mean OHC and IHC densities (hair cells/μm) in DS mice are similar to those in WT mice over the apical 5,250 μm of the cochlea, but the cochlea is longer in WT mice so that OHCs and IHCs are only present from 5,250 to 5,888 μm in WT mice. Mean cochlear length (+SEM) in WT mice (*n* = 4) is significantly longer than in DS mice (*n* = 6). ^#^ *p* = 0.0147.

To determine if there were major differences in hair cell density along the length of the cochlea, we counted the numbers of OHCs and IHCs in 100 μm intervals along the length of the cochlea in DS mice (*n* = 6) and WT (*n* = 4) mice. The mean (+/− SEM) OHCs (Figure 7A) and IHCs (Figure 7B) per 100 μm were similar in DS and WT mice over the apical 5,250 μm length of the cochlea. Over this interval, the mean OHCs per 100 µm was 38.3 in WT mice and 37.8 in DS mice while the mean IHCs per 100 µm was 11.9 in WT mice and 11.7 in DS mice (*t*-test, *p* > 0.05). There was no significant difference in OHC density (t 112 = 0.994, *p* > 0.05) or IHC density (t 112 = 1.24, *p* > 0.05) between DS and WT mice. However, the mean length of the cochlea was ∼638 μm longer in WT than DS mice (Figure 7C). The mean (+SEM, *n* = 4) length of the WT cochlea (5,888 μm) was significantly longer (*t*-test, t 3, 5 = 6.065, *p* < 0.0147) than the mean (*n* = 6, +SEM) length (5,250 mm) of the DS cochlea (Figure 7C). Therefore, the total numbers of OHCs and IHCs were slightly greater in WT than DS mice. Based on hair cell densities near the base of the cochlea, there are approximately 350 and 60 more OHCs and IHCs in the WT cochlea compared to the DS cochlea.

### Temporal bone analysis

The lack of cochlear hair cell loss together with the flat 15–20 dB hearing loss suggested that the hearing impairment in DS mice might be caused by structural anomalies in the external and/or middle ear conductive apparatus. During removal of the temporal bones for various analyses, a gross visual inspection was made of the external ear canal and bony otic capsule. Cerumen (i.e., earwax) was present which partially obstructed the external canal in 46.7% (*n* = 15) of DS mice whereas it was never observed (0%, *n* = 15) in WT mice. Under the dissecting microscope, the round window appeared noticeably smaller in 40% of DS mice (*n* = 15) relative to WT mice (*n* = 15). The stapedial artery was located below the round window in 46.7% of DS mice (*n* = 15) versus 40.0% (*n* = 15) in WT mice. The middle ear cavity appeared smaller and the bone surrounding the otic capsule seemed thicker in DS than WT mice.

**FIGURE 8:**
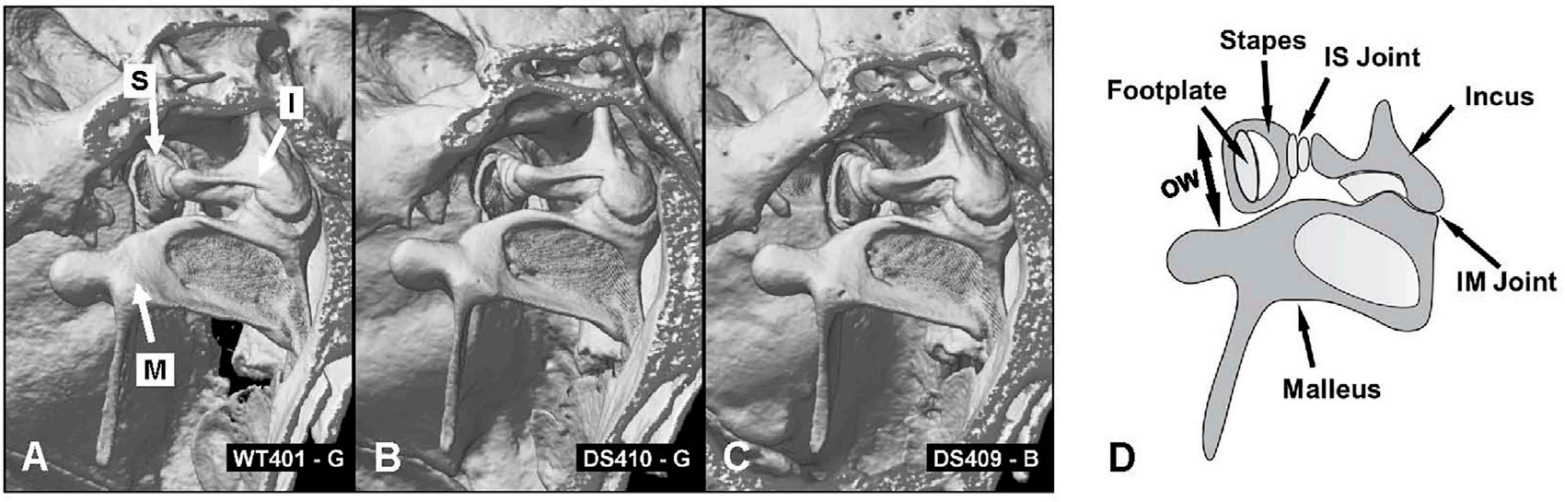
No major structural differences between ossicles in WT and DS mice. **(A)** CT image of three ossicles: stapes (S), incus (I) and malleus (M) of WT mouse compared to same structures in two DS mice, **(B)** one with near normal thresholds and another **(C)** with elevated ABR thresholds. **(D)** Schematic of three middle ear ossicles with footplate of stapes, crus of stapes, incus, malleus, incudostapedial joint (IS) and incudomalleor joint (IM). **(D)** Schematic of the ossicular chain showing the footplate of the stapes that inserts into the oval window (OW, arrow denotes maximum width of oval window), crus of the stapes, incus, malleus, incudostapedial (IS) joint connecting the head of the stapes to the incus and incudomalleor (IM) joint connecting the incus to the malleus.

To evaluate these differences in more detail, we compared micro CT images of the osseous middle ear and inner ear structures of four DS mice and two WT mice to look for major structural differences between the two genotypes (Supplementary Video S1, full structural overview). Representative micro CT images of the stapes, incus and malleus that form the ossicular chain in a WT mouse are shown in Figure 8A (Supplementary Video S2). The arrows point to head of the stapes (S), the body of the incus (I) and the malleus (M). The structure, size and orientation of the stapes, incus and malleus of a DS mouse with nearly normal ABR thresholds (Figure 8B) and a DS mouse with greatly elevated ABR thresholds (Figure 8C) were similar to those of the WT mouse (Figure 8A). No major structural differences between the ossicles of DS and WT mice were observed in our microCT images. To aid in the interpretation of the images, a schematic of the ossicular chain is presented in Figure 8D. The schematic shows the footplate of the stapes, the crus of the stapes, the incus, the malleus, the incudostapedial (IS) joint, which connects the head of the stapes to the incus, and incudomalleor (IM) joint, which connects the incus to the malleus. The footplate of the stapes is located within and surrounded by the oval window (OW). The maximum diameter of the OW (two-headed arrow) was determined from microCT images.

**FIGURE 9:**
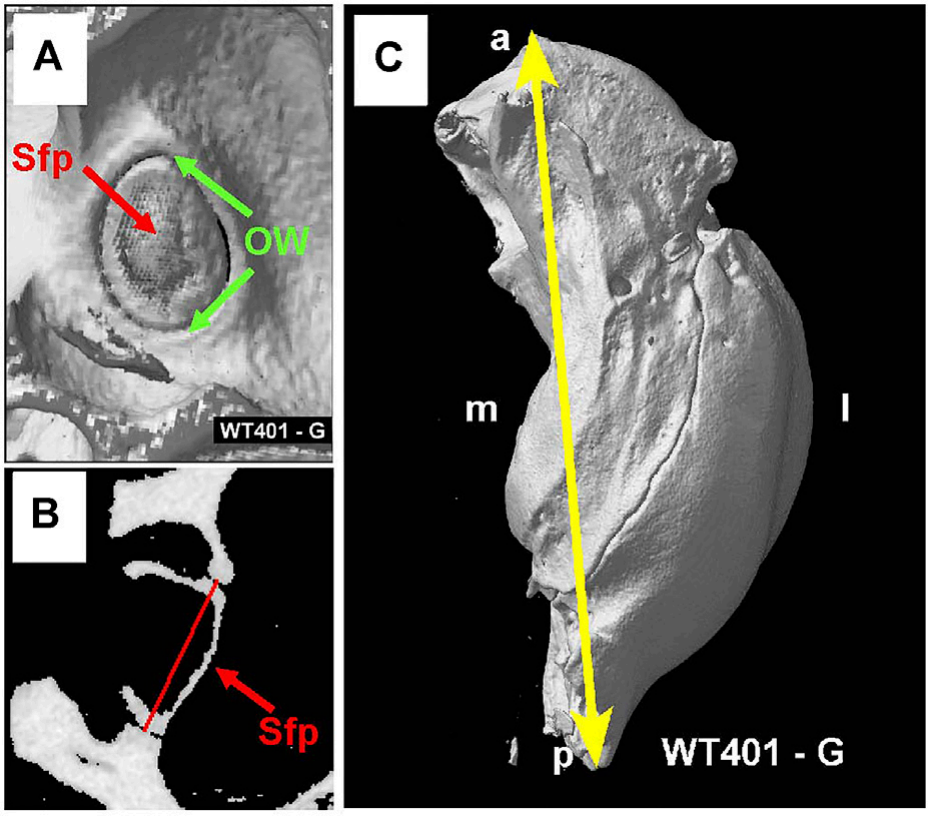
**(A)** MicroCT view showing interior surface of the stapes footplate (Sfp, red arrow) within oval window (OW, green arrows). **(B)** MicroCT cross section showing maximum width (red line) of oval window aperture. **(C)** The overall length of the tympanic cavity was measured in all samples as illustrated for a WT mouse (yellow line). Sample orientation: anterior (a), posterior (*p*), medial (m) and lateral (l).

Figure 9A shows the interior surface of the stapes footplate (Sfp, red arrow) which resides within the oval window (OW, green arrows). From the microCT images, we obtained cross sections of the footplate of the stapes within the OW. We then determined the maximum width between the bony edges of the OW (Figure 9B, red line). We also measured the maximum length of the bulla, the temporal bone cavity, which contains the cochlea and vestibular organs (Figure 9C).

**FIGURE 10:**
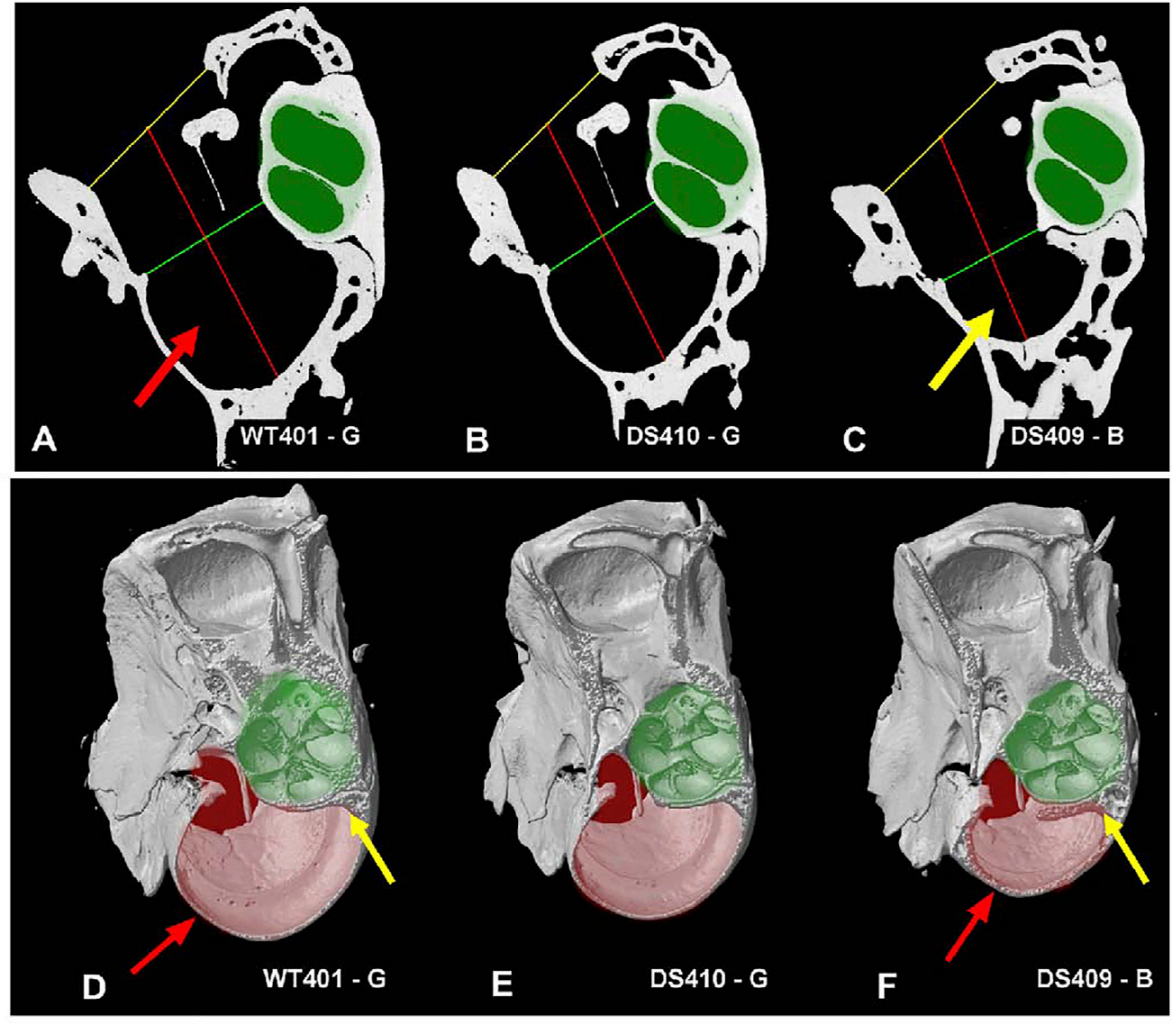
Figure illustrating tympanic cavity measurements and tympanic cavity wall thickness. Image showing measurements taken for the tympanic cavity of a **(A)** WT mouse, **(B)** a DS mouse with relatively normal ABR thresholds and **(C)** a DS mouse with greatly elevated ABR thresholds. Measurements for the tympanic membrane opening width (yellow line) and mid-tympanic cavity width (green line) were taken across the shortest distance at their respective locations. The tympanic cavity depth (red line) was taken passing through the midpoint of the two width lines. The overall dimensions of the two DS cochleae (B-C, highlighted in green) were not noticeably different from the WT mouse cochlea **(A)**. However, the tympanic cavity in the DS mouse with elevated ABR thresholds (C, yellow arrow) was visually much smaller than the tympanic cavity in the WT mouse with normal ABR thresholds (A, red arrow) and the DS mouse with nearly normal ABR thresholds **(B)**. **(D–F)** Tympanic cavity wall was often thicker in DS mice with elevated ABR thresholds than WT mice. **(D)** WT mouse with normal ABR thresholds, thin-walled temporal bone capsule (red arrow), and thin layer of bone around cochlea (yellow line). **(E)** DS mouse with relatively normal ABR thresholds had thin-walled temporal bone capsule and thin layer of bone around cochlea. **(F)** DS mouse with greatly elevated ABR thresholds had thick-walled temporal bone capsule (red arrow) and thick bone around the cochlea (yellow line). The tympanic cavity (pale-red highlight) is notably smaller in the **(F)** DS mouse with greatly elevated thresholds compared to **(D)** WT mouse with normal ABR thresholds and **(E)** DS mouse with nearly normal ABR thresholds. Cochlea (pale-green highlight) approximately the same size in **(D)** WT and **(E,F)** two DS mice.

Because the bulla, which contains the temporal bone cavity, appeared smaller than normal in some DS mice, we attempted to visualize and quantify these differences by taking microCT cross sections in a plane roughly parallel to the mid-modiolar plane of the cochlea. Cross sections of the middle ear space are shown for a WT mouse (Figure 10A), a DS mouse with nearly normal ABR thresholds (Figure 10B), and a DS mouse with greatly elevated ABR thresholds (Figure 10C, Supplementary Video S3, animations of the tympanic cavity). The interior of the cochlear capsule within the bulla is highlighted in green in each of the three specimens. From these cross sections, we measured: 1) the width of bony ring (i.e., annulus) in which the tympanic membrane resides (yellow line), 2) the mid-tympanic cavity width (shortest distance from the lateral wall of the bulla to the bony edge of the apex of the cochlea, green line), and 3) the tympanic cavity depth (distance of the red line that extends from the midpoint of the yellow line, passing through the midpoint of the green line to the wall of the tympanic cavity (bulla). Visual inspection of these three cross sections failed to reveal major differences in the size of the cochlea (green area). However, the air-filled space within the bulla (i.e., black area surround by bone) appeared noticeably smaller in the DS mouse with elevated ABR thresholds (Figure 10C) compared to the WT mouse (Figure 10A) and the DS mouse with nearly normal ABR thresholds (Figure 10B).

Mean values and ranges of the oval window diameter and other dimensions of the temporal bone cavity (Figures 10A–C) of WT (*n* = 2) and DS mice (*n* = 4) are presented in Table 1. To aid in the comparison, the mean values of the DS mice were normalized to the means in WT mice. The mean length of the middle ear cavity was 6.22 mm in DS mice compared to 6.91 mm in WT mice, approximately 10% smaller than normal. The mean width of the OW was 0.43 mm in DS mice versus 0.48 mm in WT mice, roughly 9% smaller than normal. The mean width of the tympanic membrane opening was 1.57 mm in DS mice compared to 1.73 mm in WT mice, approximately 10% smaller than normal. The mean width of the tympanic cavity at its midpoint was 1.23 in DS mice versus 1.43 mm in WT mice, about 15% below normal. Finally, the mean depth of the tympanic cavity was 2.57 mm in DS mice versus 2.94 mm in WT mice, about 13% below normal.

**TABLE 1:** Dimension of middle ear cavity (mm).

Genotype	Length middle ear cavity	Width oval window	Width tympanic membrane opening	Width midpoint tympanic cavity	Depth tympanic cavity
WT					
Mean	6.91	0.48	1.73	1.43	2.94
Range	0.10	0.04	0.03	0.06	0.11
N	2	3	2	2	2
DS					
Mean	6.22	0.43	1.57	1.23	2.57
%WT	90.1%	91.2%	90.6%	85.6%	87.3%
Range	0.61	0.03	0.10	0.18	0.49
N	4	5	4	4	4

When the tympanic cavity was removed for histological analysis of the cochlea and vestibular system, the bone surrounding the temporal bone capsule appeared to be denser in some DS mice compared to WT mice. To visualize this difference, Figures 10D–F shows microCT images taken approximately parallel to the long axis of the cochlea (Supplementary Video S4, difference images). The approximate boundaries of the cochlea are highlighted in pale green and the deep portion of the temporal bone capsule is highlighted in pale red. The bony wall surrounding the temporal bone capsule (Figure 10D, red arrows) and bone surrounding the cochlea (Figure 10D, yellow arrow) of the WT mouse and the DS mouse with nearly normal ABR thresholds (Figure 10E) were thinner than those in corresponding regions of the DS mouse with greatly elevated ABR thresholds (Figure 10F, red, yellow arrows). The tympanic cavity, which is highlighted in pale red, was noticeably smaller in the DS mouse with greatly elevated ABR thresholds (Figure 10F) compared to the WT mouse (Figure 10D) or the DS mouse with nearly normal ABR thresholds (Figure 10E).

## Discussion

### Conductive hearing loss

Patients with DS present with hearing loss of diverse origins. Many reports indicate that the hearing loss is conductive in nature caused by structural abnormalities of the external and/or middle ear (Krmpotic-Nemanic, 1970; Igarashi et al., 1977; Schwartz and Schwartz, 1978; Balkany et al., 1979; Harada and Sando, 1981; Grundfast and Camilon, 1986; Diefendorf et al., 1995; Hassmann et al., 1998; Austeng et al., 2013; Saliba et al., 2014). However, cochlear pathologies suggestive of sensorineural hearing loss have also been reported (Glovsky, 1966; Brooks et al., 1972; De Schrijver et al., 2019), including early onset presbycusis by the second decade of life (Buchanan, 1990). Still others have reported neural abnormalities in the central auditory pathway potentially contributing to central auditory processing deficits (Squires et al., 1980; Folsom et al., 1983; Widen et al., 1987). To test for these pathophysiologies, we examined the structural and functional deficits in our DS model in which we removed the confounding effect of the cadherin 23 mutation that causes early-onset, high-frequency hearing loss and hair cell loss (Kane et al., 2012; Johnson et al., 2017). DPOAE thresholds in our DS mice were elevated and DPOAE amplitudes and input/output function slopes were reduced across all frequencies (Figure 3). These DPOAE deficits resemble those observed in mice with conductive hearing loss (Qin et al., 2010). We found no evidence of hair cell loss in 9-month old DS mice that could lead to sensorineural hearing loss (Figure 7). ABR thresholds were elevated roughly 15–20 dB across all frequencies; this flat hearing loss is consistent with conductive hearing loss (Figure 4B) (Hassmann et al., 1998; Qin et al., 2010). Wave I input/output functions (Figures 4C–F) and ABR input/output functions (Figures 4G–J) provide additional support for a conductive hearing loss (Figures 4A–D). Wave I and ABR input/output functions in our DS mice were parallel to those in WT mice, but shifted to the right ostensibly due to a conductive hearing loss. Although DS reportedly accelerates age-related hearing loss in patients (Buchanan, 1990), we found no evidence of early-onset hair cell loss indicative of presbycusis in 9-month old DS mice.

### Auditory brainstem response latency and amplitude

Abnormal ABR responses have been reported in young adult patients with DS; thresholds were elevated, amplitudes were reduced and latency-intensity functions were steeper than normal putatively the results of high-frequency sensorineural hearing loss (Widen et al., 1987). Others, however, have reported shorter than normal ABR latencies in patients with DS at various developmental ages (Kaga and Marsh, 1986; McNeill and Larsen, 1988; Kittler et al., 2009). When ABR amplitudes and latencies in our DS mice were compared to their controls at the same intensity, we found that amplitudes were reduced and latencies prolonged (Figures 5A,B). However, these differences largely disappeared when a correction was made for the putative conductive hearing loss in DS mice. This was accomplished by comparing ABR responses from the two genotypes at roughly the same intensity above their respective ABR thresholds (Figures 5C–G).

### Middle ear anomalies

We did not detect any major structural abnormalities in the ossicles of DS mice (Figure 8). Our observations are consistent with the lack of significant ossicular malformation in CT images obtained from patients with DS (Saliba et al., 2014). However, our microCT results identified several structural differences in the middle ear cavity of DS mice. The width of the tympanic membrane opening is approximately 10% smaller in DS mice compared to WT littermates (Table 1). This result is consistent with the significantly smaller tympanic membrane opening observed in CT images of DS patient temporal bones (Saliba et al., 2014). The decreased diameter of the DS-mouse tympanic membrane would likely cause only a minor reduction in the acoustic energy transmitted through the ossicular chain to the stapes. The width of the oval window in which the stapes resides was roughly 9% smaller in DS mice compared to WT littermates (Table 1). This would reduce the total area of the stapes “piston” that transmits acoustic energy into the fluid filled cochlea. We are unaware of any other study reporting a decrease in the size of the oval window; however, other case reports have noted deformities in the stapes superstructure and fixation of the stapes in the oval window (Balkany et al., 1979). The length and depth of the middle ear cavity was also reduced by 10% and 13% respectively in DS mice relative to WT mice. A reduction in the total volume of the DS-mouse middle ear space would likely alter acoustic impedance of the middle ear. In contrast to our results, no major middle ear space anomalies were observed in CT images from patients with DS (Saliba et al., 2014).

### Hair cells, cochlear length and body weight

Many transgenic mice are developed on genetic backgrounds that harbor the cadherin 23 age-related hearing loss mutation. This mutation results in early, age-related high-frequency hearing loss and hair cell loss (Kazee et al., 1995; Spongr et al., 1997; Zheng et al., 1999; Johnson et al., 2010; Kane et al., 2012). To avoid this confounder, we backcrossed our Dp(16)1Yey mice (Li et al., 2007) for at least two generations on to CBA/J mice, a strain which retains normal hearing and hair cells until late in life (Spongr et al., 1997; Zheng et al., 1999; Han et al., 2016). We used sequencing to confirm that the B6-specific age-related Cdh23c753A allele was converted to the CBA/J specific Cdh23c753G allele (Kane et al., 2012). The efficacy of our approach was confirmed by the absence of cochlear hair cell loss in 9-month old DS mice (Figures 7A,B), an age at which extensive hair cell loss is typically observed in the base of the cochlea of mice with the Cdh23c735A allele (Spongr et al., 1997).

Although we did not observe a difference in hair cell densities between DS and WT mice, we found that the DS cochlea was approximately 0.6 mm shorter than in WT littermates. Because of this length difference, there are approximately 350 and 60 fewer OHCs and IHCs in the DS mouse cochlea. The shorter length of the cochlea and smaller tympanic cavity (Figure 10; Table 1) are likely related to the smaller body weight in DS mice compared to WT littermates (Figure 2). These observations are consistent with the reduced size and slow growth rates in patients with DS (Cronk et al., 1988; Styles et al., 2002).

Patients with DS exhibit balance problems (El Shennawy, 2015; Jain et al., 2022), which could be due peripheral vestibular disorders (Inagaki et al., 2011; Intrapiromkul et al., 2012; Villarroya et al., 2012; Clark et al., 2017; Capio et al., 2018). Analysis of DS patient temporal bones revealed significantly lower vestibular hair cell densities (15%–20%) in young DS subjects compared to age-matched controls (Inagaki et al., 2011). Vestibular ganglion cell densities were also significantly lower in patients with DS. We did not observed a significant difference in cochlear hair cell densities between DS and WT mice; therefore, we only conducted a cursory visual assessment of vestibular hair cell densities. We did not observe obvious differences in vestibular hair cell densities between DS and WT. Given the vestibular hair cells deficiencies noted in patients with DS, a more in depth quantitative analyses of vestibular densities should be carried out to determine if vestibular hair cells densities are below normal in young adult and/or much older DS mice.

### Limitations

Collectively, our functional and anatomical measures point to a conductive hearing impairment as the major cause of hearing loss in our DS mice. However, a more direct and informative method of quantitatively characterizing the nature of a conductive impairment would be to conduct impedance audiometry or wideband acoustic absorbance of the middle ear (Zheng et al., 2007; Ellison et al., 2012; Keefe et al., 2012; Zhang et al., 2012; Guan et al., 2017; Zheng et al., 2022). Unfortunately, the equipment needed to conduct such measurements was unavailable to us at the time when this study was conducted. These functional measures combined with detailed histopathological analysis of the external and middle ear could provide mechanistic insights on the nature of conductive loss.

Structural imaging studies have shown that many brain regions are significantly smaller in patients with DS (Fujii et al., 2017; Rodrigues et al., 2019). Major differences have been observed in the brainstem, most notably in the pons, hippocampus, frontal lobe and portions of cerebellum (Fujii et al., 2017; Hamner et al., 2018). Moreover, DS is associated with accelerated brain aging, changes that could contribute to deficits observed in the late auditory evoked potentials from adult patients with DS (Cesar et al., 2010). Taken together, these results suggest that two fruitful areas of research to pursue in DS mice would be to investigate age-dependent neuroanatomical changes in the central auditory pathway together with the assessment of higher order auditory evoked potentials that could potentially identify central auditory processing deficits. The 9-month old DS mice in our study are roughly equal in age to a 30-year old human (Dutta and Sengupta, 2016). Thus, further evaluation of our DS mice in later life could potentially reveal accelerated auditory aging in the DS central auditory pathway and other brain regions.

## Data Availability

The raw data supporting the conclusion of this article will be made available by the authors, without undue reservation.
