# Supplementary material for: Hearing impairment in murine model of Down syndrome
Source: Front Genet. 2022 Aug 4;13:936128. doi: 10.3389/fgene.2022.936128 (PMC9385999; doi:10.3389/fgene.2022.936128)
Supplement: Supplementary file 5 [file DataSheet1.docx]

Supplementary Material

# Methods

# 1.1 Scale-independent microCT Structural Difference Methods

To identify potential structural differences between DS and WT samples, independent of gross-size differences, the rigid registered microCT images were further registered to each other in a stepwise manner. First, an isotropic scaling registration was applied using the default parameters, followed by a dense vector field registration, using all default parameters, except for smoothing, which was reduced to 0.18 mm. After vector registration, image subtraction was used to generate images of the differences between DS samples and a WT sample (i.e., WT401G - DS409B, and WT404G - DS393B). In the resulting subtracted images, negative values represent areas where the DS sample has bone that the WT does not, and positive values represent areas where the WT sample has bone that the DS does not. These two subtracted images were then added together and all voxel values were cubed to highlight regions of common differences. This common differences image was then split into two channels, red for negative values, and green for positive values. The result of this is shown in Supplemental Video 4.

**1.2 Sequencing Methods**

Mouse tail tips from different mouse strains were lysed with protease at 55 °C for overnight. The lysate was further purified with phenol:chloroform:isoamyl alcohol (24:24:1) extraction and ethanol precipitation. PCR amplification of Cdh23 gene fragment with the single nucleotide variant (c753) was carried out with the purified genomic DNA and gene-specific primers, Cdh23-753M-F (CTCAGGAACACCAAAGAACC) and Cdh12-753M-R (TGAGAGAGAGGTCTCACAAC). Sanger sequencing of Cdh23 fragments was performed by the Genomic Shared Resource at RPCCC to detect the single nucleotide variant of Cdh23 allele. The sequence result from each mouse strain was shown in Supplementary Figure 1.

# Supplementary Videos and Figures

## Supplementary Videos

**Supplementary Video 1.** Overview of temporal bone. Animation showing the overall structure of the temporal bones.

Supplemental Video 2. Ossicles close-up. Animation highlighting the ossicles and their joints.

Supplemental Video 3. Tympanic cavity overview. Animation showing cross-sections of the tympanic cavity illustrating the change in volume and wall thickness.

Supplemental Video 4. Size-independent structural differences between DS and WT samples. An animation of sample DSR221409 with a two- color overlay. Green overlay indicates regions where WT samples have bone not present in corresponding regions of DS samples. Red overlay indicates regions where DS samples have bone not present in corresponding regions of WT samples.

**2.2 Supplementary Figure**

**Supplementary Figure 1.** Sequence confirmation of the strain background carrying the homozygous Cdh23^c753G^ allele in Dp(16)1Yey mice and the wild-type control mice. The tail DNA samples from a B6-Dp(16)1Yey mouse, a CBAB6F1-Dp(16)1Yey mouse and a CBAB6F2-Dp(16)1Yey mouse were purified and used as PCR template to amplify a fragment of the Cdh23 allele. Sequencing results revealed the single base change (marked by the red *) from homozygous Cdh23^c753A^ in the B6-Dp(16)1Yey mouse to the heterozygous Cdh23^c753A/G^ in the CBAB6F1-Dp(16)1Yey mouse and the homozygous Cdh23^c753G^ in the CBAB6F2-Dp(16)1Yey mouse.
